# Supplementary material for: The Association Between Adiposity and Inpatient Hospital Costs in the UK Biobank Cohort
Source: Appl Health Econ Health Policy. 2018 Dec 31;17(3):359–70. doi: 10.1007/s40258-018-0450-2 (PMC6535149; doi:10.1007/s40258-018-0450-2)
Supplement: Supplementary file 1 — Supplementary material 1 (DOCX 74 kb) [file 40258_2018_450_MOESM1_ESM.docx]

*APPLIED HEALTH ECONOMICS AND HEALTH POLICY*

**The association between adiposity and inpatient hospital costs in the UK Biobank cohort**

Padraig Dixon^1,2^*, George Davey Smith^1,2,3^, William Hollingworth^1^

1: Population Health Sciences, Bristol Medical School, University of Bristol, Bristol, BS8 2PS

2: MRC Integrative Epidemiology Unit, Oakfield House, Oakfield Grove, University of Bristol, Bristol, BS8 2BN

3: NIHR Biomedical Research Centre, Oakfield House, Oakfield Grove, University of Bristol, Bristol, BS8 2BN

*Corresponding author

Email: padraig.dixon@bristol.ac.uk

This supplementary file contains details of:

- Tests of family and link functions for generalised linear models (GLMs)
- Use of private health insurance in the Biobank cohort
- Key assumptions made in creating the cost data
- A further representation of sensitivity analysis for the continuous cost outcome
- Relative effects for categorical BMI

# Annex 1 Choice of family and link functions for generalised linear models.

The main text noted that a variety of tests were used to inform the choice of family and link functions for generalised linear models (GLM). No test on its own is necessarily definitive, and robust standard errors in all reported models were used to account for possible mis-specification.

- Box-Cox test for link function

The estimated theta coefficient from the covariate-adjusted Box-Cox model on non-zero cost observations was -0.10 for the continuous BMI exposure. A value close to zero suggests a natural logarithm transformation, whereas a value closer to 0.5 suggests a square root transformation may be appropriate, while a value close to 1 suggests the use of a linear model.

- Pregibon test for link function

The Pregibon test was estimated using the -linktest- command in Stata. The “hat squared” term in the modified regression was not significant at a 1% level with a p-value of 0.138, which suggests that the link tested should not be introduced.

- Modified Park test for family

The estimated coefficient from the modified Park test for all specifications of the outcome variable (continuous, categorical and binary) was 1.7. A value close to 2 indicates that a Gamma family is appropriate, a value close to 1 suggests the use of a Poisson distribution, and a value of zero suggests the use of a Gaussian distribution.

The use of information criteria (e.g. Akaike Information Criteria and Bayesian Information Criteria) to inform family/link function choice is not recommended as the comparisons are not necessarily meaningful across models with different families and link functions.

The results of all conducted tests suggested that a GLM with gamma family and log-link was likely to be appropriate for these data.

# Annex 2 Private health insurance

At the baseline appointment, participants were asked “Do you use private healthcare”. Table A2 summarises responses for 169, 623 individuals represented in the analysis sample.

Table A2 Use of private healthcare

| **“Do you use private healthcare”** | **N** | **Percentage of all responses** |
| --- | --- | --- |
| Yes, all of the time | 4,067 | 2.4% |
| Yes, most of the time | 4.754 | 2.8% |
| Yes, sometimes | 40,883 | 24.1% |
| No, never | 119,955 | 70.7% |

Figure A3 shows how the probability of having non-zero inpatient costs varies by reported use of private healthcare.

Figure A3 Use of healthcare

Patients who reported exclusive use of private healthcare nevertheless have a non-zero probability of reporting NHS inpatient hospital costs. In part, this will be because these costs include the costs of private patients treated in NHS hospitals, which is reflected in NHS hospital episode data. Changes in the use of private healthcare after the baseline appointment is another possible explanation, although frequencies of response to the same question concerning private healthcare were similar in a follow-up question asked of 20,339 participants at repeat assessment visits that took place in 2012 and 2013.

# Annex 3 Assumptions made in creating cost data

**Scottish care providers**

It was not possible, using the data available at the time of analysis, to distinguish between the source of specific inpatient care episodes. We therefore excluded participants who were recruited at Scottish assessment centres, or who reported any care recorded in Scottish inpatient care records.

**Episode status**

Only completed episodes were included in analysis, i.e. if the “Episode status” field was coded as “3”.

**Duplicates**

Duplicates in the hospital records data were identified as having the same patient identifier, episode start date, episode order, episode end data, admission date, discharge date, treatment and main speciality codes, operations and procedures and diagnoses.

**Patient age for each episode**

Patient age reported at the baseline Biobank appointment was updated to correspond to episode start dates. Patient age after the year of the baseline appointment is not necessarily precise in all cases, as only month and year of birth are reported in Biobank to prevent identifiability. This lack of precise age will only affect a small number of cohort participants, and is unlikely to have materially changed attribution of costs in any case.

**Recoding of inpatient record fields**

“Treatment speciality” was recoded to “General Medicine” when this field was missing or incorrectly coded.

“Main speciality” was recoded to “General Medicine” where this field was missing or incorrectly coded, except in cases where old Gastroenterology fields where indicated, in which case it was recoded to a Gastroenterology field.

“Patient classification” was recoded to “Ordinary admission” when this field was missing, or where an episode was recorded incorrectly as a Regular day or night attender admission.

“Source of admission” was recoded to “Not known” when missing. A small number of fields reflected older codes for maternity care and for mental health/learning disabilities, and these were recoded to reflect updated codes.

“Method of admission” was recoded to “Not known where missing, and otherwise updated where necessary, including to reflect a change in the coding of this variable in the 2013/14 financial year.

“Destination on discharged” was recoded to “Not known where missing, and otherwise updated where necessary.

“Episode duration” was calculated from start and end dates where not present, and reflected recoding of dates in some cases, e.g. where start date was after end date.

Where necessary ICD-10 diagnoses were recoded to the nearest general diagnosis in the same subchapter, and similarly for OPCS-4 procedure codes.

# Annex 4 Further sensitivity analysis

Figure A4 summarises average adjusted predicted cost for the sensitivity analyses described in the main text in the Results section.

**Figure A4 Averaged adjusted predicted cost per person year for continuous BMI**

It indicates the all models are similar to the base specification over most or all of the the distribution of BMI, with the exception of the analysis excluding individuals reporting any existing conditions at baseline.

# Annex 5 Relative effects of categorical BMI

Table A5 summarises relative effects of categorical BMI on inpatient costs, relative to the base category of BMI 25kg/m2 to 27.5kgm2.

Table A5 Relative effects of categorical BMI on inpatient costs

| **BMI category** | **Relative effect** | **99% confidence interval** |
| --- | --- | --- |
| 10 to <18.5 kg/m2 | 9.6% | -14.8% to 41.1% |
| 18.5 to <20 kg/m2 | -7.4% | -15.5% to 15.% |
| 20 to <22.5 kg/m2 | -9.5% | -13.0% to -5.8% |
| 22.5 to <25 kg/m2 | -4.8% | -7.9% to -1.7% |
| 25 to <27.5 kg/m2 (Base category) | - | - |
| 27.5 to <30 kg/m2 | 10.7% | 7.2% to 14.2% |
| 30 to <35 kg/m2 | 24.6% | 20.9% to 28.4% |
| 35 to <40 kg/m2 | 43.8% | 37.4% to 50.5% |
| >= 40 kg/m2 | 69.5% | 59.6% to 80.1% |
